# Papel do Endotélio na COVID-19 Grave

**DOI:** 10.36660/abc.20200643

**Published:** 2020-12-01

**Authors:** Simone Cristina Soares Brandão, Emmanuelle Tenório Albuquerque Madruga Godoi, Júlia de Oliveira Xavier Ramos, Leila Maria Magalhães Pessoa de Melo, Luca Terracini Dompieri, Djair Falcão Brindeiro, Emanuel Sávio Cavalcanti Sarinho

**Affiliations:** 1 Universidade Federal de Pernambuco RecifePE Brasil Universidade Federal de Pernambuco, Recife, PE - Brasil; 2 Serviço de Hematologia de São José dos Campos São José dos CamposSP Brasil Serviço de Hematologia de São José dos Campos, São José dos Campos, SP - Brasil

**Keywords:** Doenças Cardiovasculares/fisiopatologia, COVID-19, Betacoronavirus, Endotélio, Imunidade, Aterosclerose, Trombose

## Introdução

Estudos têm revelado uma relação significativa entre a gravidade da COVID-19 ( ***CO*** *rona* ***VI*** *rus* ***D*** *isease* 2019) e marcadores imunes. Sabe-se, por exemplo, que o endotélio participa ativamente da resposta imune e interage intimamente com o sistema de coagulação.^1^ Além disso, processos inflamatórios crônicos do endotélio estão envolvidos na fisiopatologia das doenças cardiovasculares (DCV) e metabólicas.^2^ Essas afecções podem impactar negativamente na evolução da COVID-19, e a resposta imune exacerbada do endotélio parece ser o fator determinante desse efeito.^3^

O coronavírus 2 da síndrome respiratória aguda grave (SARS-CoV-2) causa infecção por meio da ligação da proteína S ao receptor da enzima conversora de angiotensina 2 (ECA-2) na superfície da célula humana.^3 - 5^ Desse modo, é observada uma redução na disponibilidade dessa enzima, amplamente expressa em vários tecidos do corpo humano, notadamente em pulmões, coração e endotélio, com distúrbio na modulação do sistema renina-angiotensina-aldosterona (SRAA).^6^ Consequentemente, há um favorecimento da maior concentração de angiotensina 2 com uma série de ações deletérias ao organismo. Condições associadas à disfunção crônica do endotélio, como idade, hipertensão arterial sistêmica (HAS), DCV, diabetes melito e obesidade, são mais frequentes nos pacientes com COVID-19 grave ( Figura 1 ).^2 , 7^

**Figura 1:**
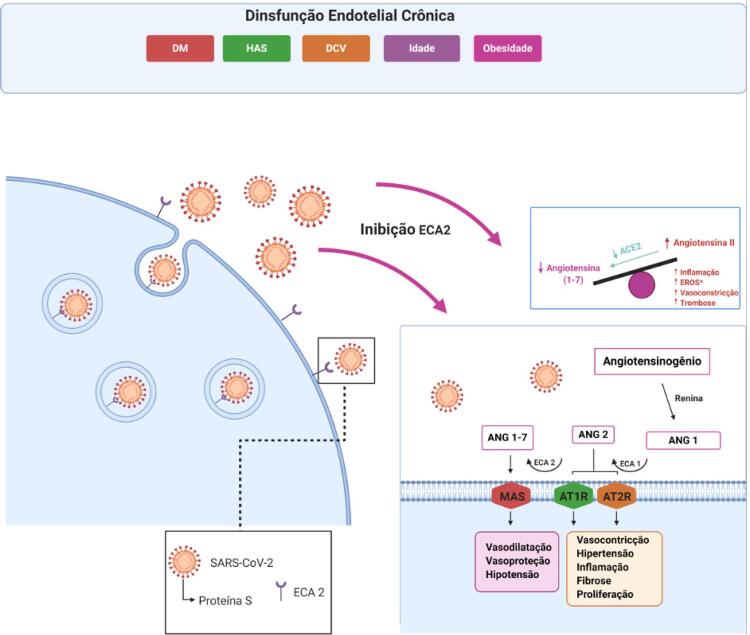
– Consequências da ligação do coronavírus 2 da síndrome respiratória aguda grave (SARS-CoV-2) com o receptor da enzima conversora de angiotensina 2 (ECA-2). A proteína S do vírus se liga ao receptor da ECA-2 da célula humana, reduzindo sua atividade enzimática. A ECA-1 e a ECA-2 agem nas angiotensinas (ANG) 1 e 2, respectivamente. A hipofunção da ECA-2 leva a uma diminuição na concentração de ANG 1-7 e, consequentemente, a um aumento na quantidade de ANG 2, com efeitos deletérios a órgãos e tecidos. Comorbidades como diabetes melito, HAS, DCV, idade avançada e obesidade causam disfunção endotelial crônica, que é agravada pela desregulação do sistema renina-angiotensina-aldosterona ocasionada pelo SARS-CoV-2. DCV: doenças cardiovasculares; EROS: espécies reativas de oxigênio; HAS: hipertensão arterial sistêmica; AT1R: receptor 1 de ANG 2; AT2R: receptor 2 de ANG 2; R-MAS: receptor de angiotensina 1-7. Fonte: elaborada pelos autores. Criada com biorender.com

Esse desequilíbrio no SRAA contribui para um estado pró-inflamatório, pró-oxidativo, com recrutamento macrofágico, excesso de citocinas circulantes, aumento na liberação de aldosterona, lesão tecidual e disfunção de múltiplos órgãos, característicos da forma grave da COVID-19.^6 , 8 , 9^ Todas essas alterações desencadeadas pelo SARS-CoV-2 podem prejudicar a função endotelial; por isso, comorbidades ligadas ao endotélio conferem maior gravidade à doença. Percebe-se, assim, na fisiopatogenia da COVID-19, uma inter-relação entre fatores pró-inflamatórios e pró-trombóticos, tornando-os importantes alvos terapêuticos.^1 , 3 , 8 - 10^

### Resposta do Endotélio à COVID-19

O endotélio desempenha papel fundamental na resposta à infecção. Isso porque células endoteliais liberam substâncias solúveis, as quimiocinas, que atraem os leucócitos para o local infectado e produzem citocinas, que ativam a resposta inflamatória. Desse modo, pacientes com disfunção endotelial crônica apresentam alterações importantes no glicocálix, nas junções intercelulares e células endoteliais, o que resulta em maior adesão e extravasamento de leucócitos, induzindo um estado de hipercoagulabilidade e redução na ação fibrinolítica. A disfunção endotelial crônica contribui, então, para o desenvolvimento da COVID-19 grave.^8 , 9^

O endotélio é um órgão ativo, indispensável para a regulação do tônus e manutenção da homeostase vascular.^1^ Na COVID-19, o recrutamento de células imunes, seja pela agressão viral direta ao endotélio ou imunomediada, pode resultar em disfunção endotelial generalizada, associada à apoptose.^3 , 11 - 13^ Estudos histológicos *post-mortem* revelaram um quadro de endotelite linfocítica em pulmões, coração, rins e fígado, bem como necrose celular e presença de microtrombos, que, nos pulmões, agravam a insuficiência respiratória.^1 , 6 , 8 , 9 , 14 , 15^

O endotélio já foi estudado em outras doenças virais, como no vírus da imunodeficiência humana (HIV) e na influenza. Assim como o HIV, o SARS-CoV-2 parece ter um efeito direto de agressão endotelial.^13 , 16^ Em estudos de autópsias, foram encontradas evidências de agressão viral direta do SARS-CoV-2 à célula endotelial e inflamação difusa.^1 , 12^ Ackermann et al.^1^ demonstraram uma quantidade de microtrombos nove vezes maior em pulmões de pessoas com COVID-19 do que naquelas com influenza. Nesses mesmos pulmões, a neoangiogênese também foi 2,7 vezes mais prevalente na COVID-19 do que na influenza.

A ideia de que estados inflamatórios crônicos subclínicos sejam responsáveis pela instalação de doenças ou pelo seu agravamento encontra-se bem estabelecida. A associação de células inflamatórias e seus respectivos produtos é bem reconhecida na fisiopatologia da aterosclerose, condição com grande repercussão no endotélio e nos componentes da síndrome metabólica (obesidade, diabetes melito e has).^11 , 17^

Embora as doenças cardiometabólicas possam iniciar na infância, é nas fases adulta e senil que ela é expressivamente mais prevalente. Na aterosclerose, assim como na COVID-19, existe predominância de resposta T_H_1, envolvendo o interferon-gama (IFN-γ), o fator de necrose tumoral alfa (TNF-α) e o beta (TNF-β), que amplificam a resposta inflamatória. O IFN-γ é considerado uma das principais citocinas pró-aterogênicas, pois ativa macrófagos e favorece a participação deles na resposta inflamatória.^11 , 12^ Torna-se evidente que uma amplificação do processo aterosclerótico ocorre a partir de uma resposta imune específica, com produção de citocinas da via T_H_1, como a interleucina-12 (IL-12) e o IFN-γ.^11 , 12^

Uma vez que o desequilíbrio no sistema imunológico está presente na fisiopatologia das DCV e da síndrome metabólica, as pessoas com essas doenças, até mesmo as mais jovens, com aterosclerose incipiente, estariam mais susceptíveis à forma grave da COVID-19, por já possuírem um “terreno” imune hiperativo e desregulado.^5 , 11 , 18^

Outra explicação para as doenças cardiometabólicas serem fatores de risco para a forma grave da COVID-19 envolve os receptores de reconhecimento de patógenos *toll-like-4* (TLR4), integrantes moleculares da imunidade inata.^5 , 18^ Já se sabe que os TLR4 participam da patogênese das DCV e metabólicas, como aterosclerose, diabetes e obesidade. Eles são expressos em diferentes tipos de células da placa aterosclerótica, e vários ligantes pró-aterogênicos podem ativá-los. Os TLR4 estão também envolvidos na lipotoxicidade e na disfunção de células betapancreáticas. A hiperexpressão dos TLR4 pode ser, inclusive, geneticamente codificada.^5 , 18^

Na imunopatologia da COVID-19 ocorre elevação, principalmente, da interleucina-6 (IL-6) e do TNF-α. Essas citocinas são produtos de ativação do TLR4. Em um estudo por simulações computacionais, demonstrou-se que a proteína S do SARS-CoV-2 é reconhecida pelos TLR4.^5^ Assim, indivíduos com maior expressão desses receptores, uma vez infectados pelo SARS-CoV-2, sofreriam maior ativação e liberação de IL-6 e TNF-α, condição vista na forma grave da COVID-19.

Como já comentado, outro provável mecanismo responsável pela pior evolução da COVID-19 envolve o receptor da ECA-2.^19^ A redução da sua atividade pelo SARS-CoV-2 tem implicações nas DCV por potencializar a desregulação do SRAA e do sistema imune.^6 , 20^ Já existem evidências de que o uso de medicações que bloqueiam o SRAA, como inibidores de ECA-1 (IECA) e bloqueadores do receptor da angiotensina (BRA), não se relaciona com aumento de mortalidade pela COVID-19, podendo inclusive ser fator de proteção.^19 , 21^

### Alterações na Coagulação na COVID-19

Estados inflamatórios exacerbados culminam em estase sanguínea, ativação plaquetária e disfunção endotelial, elevando as chances de episódios trombóticos venosos e arteriais. A coagulopatia na infecção grave por COVID-19 é semelhante à coagulopatia induzida pela sepse, caracterizada por coagulação intravascular disseminada e microangiopatia trombótica. Somado a isso, destaca-se que a hipoxemia, secundária à lesão pulmonar causada pela COVID-19, é fator de risco para trombose.^8 , 9 , 17^

O SARS-CoV-2 provoca a síndrome respiratória aguda grave (SARS), na qual ocorre acúmulo de fibrina insolúvel no espaço alveolar. Aventa-se que o fibrinogênio extravase do plasma por aumento da permeabilidade vascular e dano alveolar difuso, com eliminação incompleta devido a um estado de hipofibrinólise. Cronicamente, essa fibrina insolúvel contribui para fibrose pulmonar e seus desdobramentos negativos.^8 , 9 , 15 , 17^

As principais alterações na coagulação presentes na COVID-19 são: elevação do dímero-D, do fibrinogênio e do tempo de protrombina, e diminuição da fibrinólise. A contagem de plaquetas pode estar reduzida nos estágios mais avançados da doença, sendo fator preditivo de mortalidade.^8 , 9 , 15 , 17^ O aumento do risco de trombose ocorre também nas artérias, e diferentes manifestações clínicas podem aparecer, como: acidente vascular encefálico, isquemia mesentérica, infarto agudo do miocárdio e oclusão arterial de membros inferiores, a depender do leito arterial acometido.^22^ Corroborando a hipótese de agressão vascular, alguns casos com características da síndrome do choque tóxico ou síndrome inflamatória multissistêmica pediátrica similar à doença de Kawasaki têm sido descritos e relacionados à COVID-19.^20^

### Estratégias Terapêuticas

Considerando o exposto, destaca-se a importância do controle rigoroso dos fatores de risco cardiometabólicos.^5^ O objetivo é deixar o endotélio menos reativo e menos vulnerável à COVID-19. A otimização do tratamento medicamentoso com o uso de hipoglicemiantes, anti-hipertensivos, hipolipemiantes (principalmente as estatinas) e antiagregantes plaquetários (como o ácido acetilsalicílico) pode estabilizar o endotélio.^5 , 20 , 23^ Fármacos como IECA e BRA parecem fundamentais na redução do risco de desfechos graves pela COVID-19, uma vez que ajudam a equilibrar o SRAA.^19^ Em relação ao SARS-CoV-2, até o momento, não existe tratamento específico que seja comprovadamente eficaz no combate ao vírus. A estratégia terapêutica tem se baseado no reconhecimento precoce das complicações e no suporte otimizado para aliviar os sintomas ( Figura 2 ).

**Figura 1:**
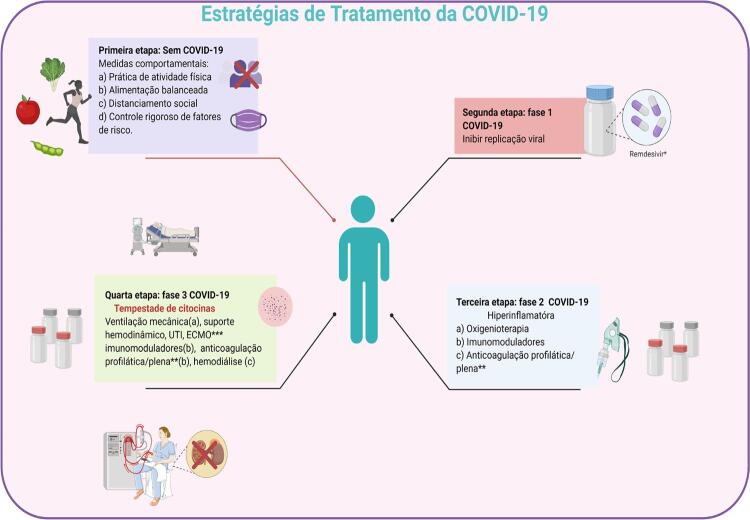
– Estratégias de tratamento para a prevenção da COVID-19 de acordo com as fases da doença. Fonte: elaborada pelos autores. Criada com biorender.com

Na fase hiperinflamatória da COVID-19, as medicações que inibam ou reduzam os efeitos das citocinas pró-inflamatórias são muito pertinentes e devem ser levadas em consideração. Os inibidores de IL-6, assim como os glicocorticoides, poderiam evitar ou amenizar a tempestade de citocinas.^23^ Novas medicações moduladoras da resposta inflamatória são fundamentais nessa fase para evitar a inflamação excessiva, que agride intensamente o endotélio e os diversos órgãos, podendo culminar com falência de múltiplos órgãos até a morte.

Em relação ao tromboembolismo venoso, os pacientes hospitalizados devem receber tromboprofilaxia farmacológica com heparina de baixo peso molecular ou fondaparinux (preferencialmente à heparina não fracionada), a menos que o risco de sangramento exceda o de trombose, quando então se faz a profilaxia mecânica.^8 , 9 , 15 , 17^ O ajuste da dose da heparina de acordo com o índice de massa corpórea e com o *clearance* de creatinina é recomendado.^8 , 15^ A heparinização plena é indicada em casos de forte suspeita clínica ou já confirmados de tromboembolismo venoso.^8 , 15^

A heparina é uma medicação já largamente utilizada na medicina, pela ação anticoagulante e pelo efeito anti-inflamatório. Entretanto, na COVID-19, além desses efeitos, estudos têm sugerido seu uso como modo de impedir a replicação viral. O SARS-CoV-2 se liga ao receptor da ECA-2 para penetrar na célula humana e se multiplicar. Acredita-se que, para essa ligação, o vírus também precisa se ligar ao heparan sulfato presente, dentre outros sítios, na membrana basal do endotélio. A utilização de heparina tem sido sugerida como estratégia de ligação ao heparan sulfato, impedindo a ligação do SARS-CoV-2 ao receptor da ECA-2 e diminuindo, assim, a replicação viral.^24^

## Conclusão

Em resumo, ressalta-se que a função endotelial é fator fundamental na progressão dos estágios clínicos da COVID-19, pois a disfunção crônica do endotélio, que acontece nas doenças pré-existentes, favorece diretamente a evolução para a forma grave da doença. Assim, enquanto a vacina é aguardada, os alvos terapêuticos ( Tabela 1 ) devem ser: controle das condições cardiovasculares, metabólicas e endoteliais da população de risco e nos infectados; e redução da replicação viral, da hiperinflamação e da hipercoagulabilidade.

**Tabela 1:** – Potenciais alvos terapêuticos de drogas candidatas ao combate da COVID-19. Como se trata de uma doença pandêmica, as etapas de tratamento devem acontecer mesmo antes das pessoas serem infectadas pelo vírus. *Ensaios clínicos já publicados não mostraram benefícios.

Etapas	Potenciais alvos terapêuticos	Medicações
Etapa 1: sem COVID-19	Anti-hipertensivos	Inibidores da enzima conversora de angiotensina (ECA) e bloqueadores dos receptores de angiotensina (BRA), principalmente
	Estatinas	Sinvastatina, rosuvastatina, atorvastatina etc.
	Antiplaquetários	Ácido acetilsalicílico
	Vacinas	Múltiplas candidatas. Pesquisas em andamento
Etapa 2: fase 1 da COVID-19	Entrada pelo receptor da ECA-2	ECA-2 recombinante solúvel
	TMPRSS2 protease S priming	Inibidor de protease (mesilato de camostato)
	Endocitose do receptor	Cloroquina ou hidroxicloroquina*
	RNA polimerase para replicação	Remdesivir, Favipiravir
	Proteases virais	Lopinavir/Ritonavir*
	Transporte nuclear pela importina	Ivermectina
Etapas 3 e 4: Fase 2 da COVID-19, hiperinflamatória Fase 3 da COVID-19, “tempestade” de citocinas	Antivirais/anti-inflamatórios	Plasma convalescente de pacientes com a COVID-19, Interferon tipo I, imunoglobulinas, células troncomesenquimais
	Ativação pelo excesso de interleucina-1	Anakinra, canaquinumabe, colchicina
	Tempestade de citocinas	Tocilizumabe, sarilumabe, siltuximabe (inibidores de interleucina-6) ou baricitinibe (inibidor da JAK), lenzilumab (inibidor do fator estimulante de colônias de granulócito-macrófago)
	Infecção bacteriana/inflamação	Azitromicina e outros antibióticos
	Coagulopatia	Regime de anticoagulação plena ou profilática
	Antivirais/anti-inflamatórios	Plasma convalescente
	Estresse oxidativo	Vitamina C, deferoxamine

*ECA 2: enzima conversora da angiotensina 2. TMPRSS2: transmembrana protease serina 2. Fonte: modificada da referência 10.*
