# Supplementary material for: Papel do Endotélio na COVID-19 Grave
Source: Arq Bras Cardiol. 2020 Dec 1;115(6):1184–9. [Article in Portuguese] doi: 10.36660/abc.20200643 (PMC8133713; doi:10.36660/abc.20200643)
Supplement: Translation

# Translation: The Role of the Endothelium in Severe COVID-19

**Authors:** Simone Cristina Soares Brandão, Emmanuelle Tenório Albuquerque Madruga Godoi, Júlia de Oliveira Xavier Ramos, Leila Maria Magalhães Pessoa de Melo, Luca Terracini Dompieri, Djair Falcão Brindeiro, Emanuel Sávio Cavalcanti Sarinho

**Affiliations:** 1 Brazil Universidade Federal de Pernambuco, Recife, PE - Brazil; 2 Brazil Serviço de Hematologia de São José dos Campos, São José dos Campos, SP - Brazil

**Keywords:** Cardiovascular Diseases/physiopathology, COVID-19, Betacoronavirus, Endothelium, Immunity, Atherosclerosis, Thrombosis

## Introduction

Studies have unveiled a significant link between the severity of COVID-19 ( ***CO*** *rona* ***VI*** *rus* ***D*** *isease* 2019) and immune markers. It is well-known that the endothelium participates actively in the immune response and interacts closely with the coagulation system.^1^Chronic inflammatory processes of the endothelium are involved in the physiopathology of cardiovascular diseases (CVDs) and metabolic diseases.^2^These diseases can negatively impact the evolution of COVID-19 and an exacerbated immune response of the endothelium seems to be a determining factor of this effect.^3^

Coronavirus 2 of the Severe Acute Respiratory Syndrome (SARS-CoV-2) causes infection by means of the link of the S protein to the Angiotensin-Converting Enzyme 2 (ACE 2) on the surface of human cells.^3^In this sense, a reduction in the availability of this enzyme can be observed, which is widely expressed in a number of bodily tissues, most notably, the lungs, heart, and endothelium, with a disorder in the modulation of the renin-angiotensin-aldosterone system (RAAS).^6^Consequently, what can be seen is a favoring of the greater concentration of angiotensin 2 with a series of deleterious actions against our organism. Conditions associated with the chronic dysfunction of the endothelium, such as age, systemic arterial hypertension (SAH), CVD, diabetes, and obesity are the most common in patients with severe COVID-19 ( Figure 1 ).^2 , 7^

**Figure 1:** – Consequences of the connection of coronavirus 2 of the severe acute respiratory syndrome (SARS-CoV-2) to the ACE 2 receptor (angiotensin-converting enzyme 2). The S protein of SARS-CoV-2 connects to the ACE 2 receptor of the human cell, reducing its enzymatic activity. The ACE 1 (angiotensin-converting enzyme 1) and ACE 2 act in the angiotensin I (ANG I) and II (ANG II). The hypofunction of the ACE 2 leads to a reduction in the concentration of angiotensin 1-7 (ANG 1-7) and, consequently, to an increase in the quantity of ANG II, with deleterious effects to the organs and tissues. Comorbidities, such as DM, SAH, CVD, advanced age, and obesity, cause chronic endothelial dysfunction, which can be aggravated by the renin-angiotensin-aldosterone system, caused by SARS-CoV-2. DM: diabetes mellitus; CVD: cardiovascular diseases; RSO: reactive species of oxygen; SAH: systemic arterial hypertension; R1AT: receptor 1 of ANG II; R2AT: receptor 2 of ANG II; R-MAS: receptor of angiotensin 1-7. Source: Figure drafted by the authors. Created with biorender.com

This imbalance in the RAAS contributes to a pro-inflammatory, pro-oxidative state, with macrophage recruitment, an excess of circulating cytokines and increase in the release of aldosterone, tissue damage, and the dysfunction of multiple organs, all characteristic of the severe form of COVID-19.^6 , 8 , 9^All of these alterations triggered by SARS-CoV-2 can harm the endothelial function. Therefore, comorbidities linked to the endothelium confer a greater severity to the disease. In the physiopathogenesis of COVID-19 one can thus observe an interaction between the pro-inflammatory and the pro-thrombotic factors, making them important therapeutic targets.^1 , 3 , 8 - 10^

### Endothelial Response to COVID-19

The endothelium plays an important role in the response to infection. Endothelial cells release soluble substances, chemokines, which attract leucocytes to the infected location and produce cytokines, in turn activating the inflammatory response. Patients with chronic endothelial dysfunction present important alterations in the glycocalyx, intercellular junctions, and endothelial cells, which results in greater leukocyte adhesion and extravasation, as well as leads to a state of hypercoagulability and reduction in the fibrinolytic action. Chronic endothelial dysfunction thus contributes to the development of severe COVID-19.^8 , 9^

The endothelium is an active organ, which is essential for the regulation of the tonus and the maintenance of vascular homeostasis.^1^In COVID-19, the recruitment of immune cells, be it through direct or immune-mediated viral aggression, can result in a widespread endothelial dysfunction, associated with apoptosis.^3 , 11 - 13^Post-mortem histological studies have demonstrated a medical picture of lymphocytic endothelialitis in the lungs, heart, kidneys, and liver, as well as cell necrosis and the presence of microthrombi, which aggravate respiratory insufficiency in the lungs.^1 , 6 , 8 , 9 , 14 , 15^

The endothelium has already been studied in other viral diseases, such as in the human immunodeficiency virus (HIV) and influenza. Much like HIV, SARS-CoV-2 also appears to be directly impacted by endothelial agression.^13 , 16^Autopsy studies have found evidence of direct viral aggression from SARS-CoV-2 against the endothelial cell, coupled with widespread inflammation.^1 , 12^Ackermann et al.^1^demonstrated a quantity of microthrombi that is nine times greater in the lungs of COVID-19 patients than in those patients with influenza. In these same lungs, the neo-angiogenesis was also 2.7 times more prevalent in COVID-19 than in influenza.

The idea that subclinical chronic inflammatory states are responsible for the installation of diseases or for their worsening is well-defined in the literature. The association of inflammatory cells and their respective products is well-known in the physiopathology of atherosclerosis, a condition which has a great repercussion upon the endothelium and in the components of the metabolic syndrome (obesity, diabetes mellitus, and hypertension).^11 , 17^

Although the cardiometabolic disease can begin during childhood, it is in the adult and senile stage that it is more expressively prevalent. In atherosclerosis, as well as in COVID-19, there is a predominance of the T_H_1 response, involving the interferon-gamma (IFN-γ), tumor necrosis factor alpha (TNF-α), and tumor necrosis factor beta (TNF-β), which amplifies the inflammatory response. The IFN-γ is considered to be one of the main pro-atherogenic cytokines, as it activates the macrophages and favors the participation of these in the inflammatory response.^11 , 12^It thus becomes evident that an amplification in the atherosclerotic process occurs as of a specific immune response, with the production of T_H_1 cytokines, such as interleukin-12 (IL-12) and IFN-γ.^11 , 12^

Since the imbalance in the immune system is present in the physiopathology of CVDs and the metabolic syndrome, people with these diseases, even younger people with incipient atherosclerosis, would be more susceptible to the most severe form of COVID-19, as they already have a hyperactive and uncontrolled immune “terrain”.^5 , 11 , 18^

Another explanation for cadiometabolic diseases being risk factors for the severe form of COVID-19 involves the recognition receptors of the *Toll-Like-4* (TLR4) pathogens, which are molecular members of innate immunity.^5 , 18^It is well-known that the TLR4 participate in the pathogenesis of the CVDs and metabolic diseases, such as atherosclerosis, diabetes, and obesity. The TLR4 are expressed in different types of cells of the atherosclerotic plaque, and various pro-atherogenic ligands can activate them. The TLR4 are also involved in lipotoxicity and in pancreatic beta cell dysfunction. The hyperextension of the TLR4 can even be genetically codified.^5 , 18^

Interleukin-6 (IL-6) and TNF-α tend to be higher in the immunopathology of COVID-19. These cytokines are products of the activation of TLR4. In a study conducted using computer simulations illustrated that the S protein of SARS-CoV-2 is recognized by the TLR4.^5^Consequently, individuals with a greater expression of these receptors, once infected by SARS-CoV-2, would suffer greater activation and release of IL-6 and TNF-α, a condition observed in the severe form of COVID-19.

As mentioned above, another probable mechanism responsible for the poor evolution of COVID-19 involves the ACE 2 receptor.^19^The reduction in the ACE 2 activity caused by SARS-CoV-2 direct influences CVDs, as they increase the potential for the deregulation of RAAS and the immune system.^6 , 20^There is already evidence that the use of medications that block the RAAS, such as ACE 1 inhibitors (ACEI) and angiotensin receptor blockers (ARBs), are not related to the increase in mortality caused by COVID-19, and can even serve as protection factors.^19 , 21^

### Alterations in COVID-19 Coagulation

Exacerbated inflammatory states culminate in blood stasis, platelet activation, and endothelial dysfunction, raising the probability of episodes of venous and arterial thrombosis. The coagulopathy in severe COVID-19 infection is similar to coagulopathy induced by sepsis, characterized by widespread intravascular coagulation and thrombotic microangiopathy. In addition, one can highlight hypoxemia, secondary to the pulmonary lesion caused by COVID-19, which is a risk factor for thrombosis.^8 , 9 , 17^

SARS-CoV-2 provokes SARS. In this syndrome, insoluble fibrin accumulates in the alveolar space. It can be said that the fibrinogen flows out of the plasma due to an increase in the vascular permeability and the widespread alveolar damage, with its incomplete elimination due to a state of hypofibrinolysis. Chronically, this insoluble fibrin contributes to pulmonary fibrosis and its negative repercussions.^8 , 9 , 15 , 17^

The main alterations in the coagulation present in COVID-19 are: rise in the D-dimer, fibrogen, and time of prothrombin, as well as a reduction in fibrinolysis. The platelet count can be reduced in the more advanced stages of the disease, and is a predictive factor of mortality.^8 , 9 , 15 , 17^The increase in the risk of thrombosis also occurs in the arteries and different clinical manifestations can appear, including: cerebrovascular accident, mesenteric ischemia, acute infarction of the myocardium, and arterial occlusion of the lower limbs, depending on the affected arterial bed.^22^Corroborating with the hypothesis of vascular aggression, some cases with features of toxic shock or pediatric multisystemic inflammatory syndrome similar to the Kawasaki disease have been described and related to COVID-19.^20^

### Therapeutic Strategies

Considering that put forth above, it is possible to highlight the importance of the strict control of cardiometabolic risk factors.^5^The aim is to render the endothelium less reactive and less vulnerable to COVID-19. The optimization of the medicinal treatment with the use of anti-diabetic, anti-hypertensive, hypolipidemic (mainly statins), and anti-platelet (such as acetyl salicylic acid) medications can stabilize the endothelium.^5 , 20 , 23^Drugs, such as ACEI and ARB, seem to be essential in the reduction of the risk of severe outcomes caused by COVID-19, since it helps to balance the RAAS.^19^Regarding to the SARS-CoV-2, to date, no specific treatment has been proven to be effective in the fight against the virus. The therapeutic strategy has been based on the early recognition of the complications in the optimized support to alleviate the symptoms ( Figure 2 ).

**Figure 2:** – Treatment strategies for prevention and according to the stages of the disease caused by coronavírus-2 (COVID-19). Source: Figure drafted by the authors. Created with biorender.com

In the hyperinflammatory stage of COVID-19, the medications that inhibit or reduce the effects of the pro-inflammatory cytokines are quite pertinent and should be taken into consideration. The IL-6 inhibitors, as well as the glucocorticoids, can avoid or lessen the storm of cytokines.^23^New modulator medications of the inflammatory response are key in this stage to avoid excessive inflammation, which intensely attacks the endothelium and the diverse organs and can result in multiple organ failure and even death.

Regarding to venous thromboembolism, hospitalized patients should receive pharmacological thromboprophylaxis with heparin in a low molecular weight or fondaparinux (preferably unfractionated heparin), unless the risk of bleeding exceeds the risk of thrombosis, in which case, mechanical prophylaxis should be applied.^8 , 9 , 15 , 17^The adjustment in the heparin dose according to the body mass index (BMI), together with the clearance of creatinine, is recommended.^8 , 15^Full heparinization is recommended in cases in which there is a strong clinic suspicion or confirmation of venous thromboembolism.^8 , 15^

Heparin is a medication that is widely used in medicine because of its anti-coagulant action and its anti-inflammatory effect. However, in COVID-19, in addition to these effects, studies have suggested the use of heparin as a way to hinder viral replication. SARS-CoV-2 connects to the ECA2 receptor in order to penetrate the human cell and multiply. It is believed that, to achieve this link, the virus also needs to connect to the heparan sulfate, among other sites, present in the endothelial membrane. The use of heparin has been suggested as a strategy of connection to heparan sulfate, hindering the connection of SARS-CoV-2 to the ECA2 receptor, thus diminishing viral replication.^24^

## Conclusion

In sum, it is important to highlight that the endothelial function is an essential factor in the progression of the clinical stages of COVID-19. The chronic dysfunction of the endothelium, which occurs in the pre-existing diseases, directly favors the evolution toward the severe form of the disease. Therefore, while we await a vaccine, the therapeutic targets ( Table 1 ) must include the control of the cardiovascular, metabolic, and endothelial conditions of the at-risk population and the infected individuals, as well as a reduction in viral replication, hyperinflammation, and hypercoagulability.

**Table 1:** – Potential therapeutic targets of potential drugs to fight COVID-19. As this is about a pandemic disease, the treatment stages should occur even before people are infected by the virus. Previously published clinical trials have shown no benefits.

Stages	Potential therapeutic targets	Medications
Without COVID-19	Anti-hipertension drugs	Mainly angiotensin-converting enzyme inhibitors and angiotensin receptor blockers.
	Statins	Simvastatin, Rosuvastatin, Atorvastatin, etc.
	Anti-platelet drugs	Acetyl-salicytic acid
	Vaccines	Multiple candidates. Research in progress.
Stage 2: Phase 1 of COVID-19	Entrance for ACE2 receptor	ACE2 soluble recombinant
	TMPRSS2 protease S priming	Protease inhibitor (camostat mesilate)
	Endocytosis of the receptor	Chloroquine or Hydroxychloroquine*
	RNA polymerase for replication	Remdesivir, Favipiravir
	Viral Proteases	Lopinavir/Ritonavir*
	Importin nuclear transportation	Ivermectin
Stages 3 and 4: Phase 2 of COVID-19 Hyperinflammation Phase 3 of COVID-19 “Storm” of cytokines	Anti-viral/anti-inflammatory	Convalescent plasma of patients with COVID-19, Interferon type I, immunoglobins, mesenchymal stem cells
	Activation through the excess of Interleukin 1	Anakinra, Canakinumab, Colchicine
	Storm of cytokines	Tocilizumab, Sarilumab, Siltuximab (IL-6 inhibitors), or baricitinib (JAK inhibitor), Lenzilumab (granulocyte- macrophage colony-stimulating fator inhibitor)
	Bacterial/inflammation infection	Azithromycon and other antibiotics
	Coagulopathy	Full and prophylactic anti-coagulation regime.
	Anti-viral/anti-inflammatory drugs	Convalescent Plasma
	Oxidative stress	Vitamin C, Deferoxamine

*ACE 2: angiotensin-converting enzyme 2. TMPRSS2: transmembrane protease, serine 2. Source: modified from reference 10
